# Measuring Explicit Prejudice and Transphobia in Nursing Students and Professionals

**DOI:** 10.3390/nursrep10020008

**Published:** 2020-10-15

**Authors:** Jesús Manuel García-Acosta, María Elisa Castro-Peraza, Lilisbeth Perestelo-Pérez, Amado Rivero-Santana, Ángeles Arias-Rodríguez, Nieves Doria Lorenzo-Rocha

**Affiliations:** 1 Canary Islands Public Health Service, Tenerife, 38071 Canary Islands, Spain; jesus.garcia.21@ull.edu.es (J.M.G.-A.); extnlorenzo@ull.edu.es (N.D.L.-R.); 2 Department of Nursing, University of La Laguna, Tenerife, 38010 Canary Islands, Spain; 3 Evaluation Unit of the Canary Islands Health Service (SESCS), 38109 Tenerife, Spain; lilisbeth.peresteloperez@sescs.es; 4 Health Services Research on Chronic Patients Network (REDISSEC), 38109 Tenerife, Spain; amado.riverosantana@sescs.es; 5 Institute of Biomedical Technologies, University of La Laguna, 38200 San Cristóbal de La Laguna, Spain; 6 Canary Islands Foundation of Health Research (FUNCANIS), 38320 San Cristóbal de La Laguna, Spain; 7 Department of Preventive Medicine and Public Health. Tenerife, 38071 Canary Islands, Spain; angarias@ull.edu.es

**Keywords:** transgender persons, transphobia, prejudice, discrimination, education, nursing

## Abstract

Trans* people frequently report attitudes of prejudice/transphobia in health professionals. Conversely, health professionals indicate the lack of adequate training to care for these people and its impact on the quality of care provided. Objective: Our objective was to evaluate the explicit prejudices/transphobia of health students and professionals and compare them with the general population in Tenerife. Methods: A descriptive cross-sectional study was carried out with the Genderism and Transphobia Scale (GTS) and the Negative Attitude towards Trans* people Scale (EANT) with a total of 602 participants. Results: We found a low mean level of explicit prejudice/transphobia, with little/no differences between occupation groups. Explicit transphobia was correlated with being a man, less educated, and heterosexual, and not personally knowing a trans* person. Men and women were less transphobic about trans* people whose identities coincided with their own. Conclusion: All participants showed a low mean level of explicit transphobia. This result is not incompatible with unconscious prejudice, which may translate to discriminatory behaviors. Interventions to change negative attitudes are still needed, since even a small percentage of transphobic health professionals could exert a considerable negative impact on health care. In professionals without transphobic attitudes, the barriers identified by trans* people might be a problem due to the lack of specific training.

## 1. Introduction

In the literature, the term trans* is accepted as a broad concept that encompasses many gender identities, especially those that do not coincide with the sex assigned at birth [1]. Therefore, many (but not all) of them want to adapt, to a greater or lesser extent, their secondary sexual characteristics by undergoing some form of gender confirmation treatment, hormone therapy, and/or surgery. The manifestation of the different gender identities depends on the sociocultural context. Different societies allow and prohibit different approaches.

Historically, the social construction of personal relationships has been determined by the principle of heteronormativity; that is, the concept of cisgender (concordance between the sex assigned at birth and gender identity), heterosexuality, and family traditionalism (i.e., other-sex marriage) as the “correct” way of living [2]. Thus, heteronormativity refers to heterosexuality being the perceived normative form for relationships and coupling. This has led to prejudice and discrimination against gender and sexual minorities, which, in the case of trans* people, has been labelled transphobia [3,4].

The term transphobia is often used in the literature [5]. Transphobia is observed and measured by different organizations. For example, Transgender Europe (TGEU), in its international campaign Transrespect vs. Transphobia Worldwide (TvT), publishes data monitoring murders of people in the diverse genders. The 2019 update shows a total of 331 hate crimes between 1 October 2017 and 30 September 2018. This means an increase in 44 cases with respect to the last update of 2017 and 76 cases compared with 2016, which adds to a total of 2982 cases reported in 72 countries all over the world between 1 June 2008 and 30 September 2019 [6]. More qualitatively, a publication from International Amnesty highlights the breadth and impact of the problem of explicit transphobia [7].

Although in the last few decades social and political changes have led to a greater acceptance of trans* people in public opinion and policies, they still suffer prejudice, discrimination, and stigma. One of the causes that can justify the presence of transphobia/prejudice is the stigma that trans* people are suffering because they must be diagnosed with a mental illness, known as “gender dysphoria” according to the DSM-V [8], to have access to trans-specific health services such as hormone therapy or surgery [9]. In most countries, it is not possible to obtain health care or change of name and sex in legal documents without a diagnosis of mental illness. In other words, pathologization is a way to obtain specific healthcare access and civil rights.

Regarding sociodemographic characteristics, research has shown that older age, male sex, religious fundamentalism, political orientation, heterosexuality, being less educated, and not knowing trans* people personally are associated with a higher level of prejudice/transphobia [5,10]. It has been observed that both cisgender men and women establish relationships without prejudice with non-heterosexual people when they do not know their sexual orientation; the same occurs with gender identity. Therefore, many trans* people hide it and remaining closeted serves as a safety mechanism [11].

Trans* people have a need for both non-trans-specific and trans-specific health care, especially those who are receiving hormone therapy, adapting their secondary sexual characteristics, or preparing for reassignment surgery. Therefore, it is important that the degree of transphobia/prejudice of health professionals be as low as possible. However, research has found that although some progress has been made, the experiences of trans* people with health services still involve discriminatory practices, with many patients reporting a lack of sensitivity and prejudices by administrative and health personnel, including nursing staff [5,10]. Some studies report negative attitudes toward sexual minorities among nursing professionals [12]. Many trans* persons are afraid of disclosing their gender identity to health professionals because of fear of hostile or insensitive reactions [3]. One study of transgender people found that 40% of trans* participants felt anxiety when they accessed health services due to stigma and potential discrimination [13]. Conversely, most health professionals recognize that they lack the necessary training, knowledge, and skills to offer adequate health care to transgender people [14]. The literature shows many experiences of educational interventions in gender diversity assistance in healthcare for undergraduate and postgraduate students, who have reported successful results in the reduction of transphobia/prejudice [10,15].

Given that most nurses feel unprepared and that they lack knowledge of the needs of trans* people [16,17,18], it is a challenge for nursing education to synthesize the growing evidence of health problems of the LGTBI collective [12]. A study revealed what happened inside nursing classrooms when there was a trans* person among the students, reporting that four out of ten trans* nursing students felt discrimination due to their gender identity within the faculty of nursing [19]. Such transphobia can have a lasting impact on their practice as a health professional [12].

Given all the aforementioned points, the aim of this study was to assess health professionals’ explicit prejudice/transphobia and to compare them with the general population (those not working in health services) in Tenerife. We also included a sample of healthcare students to explore if potential differences in attitudes are already observable at the undergraduate level.

## 2. Experimental Section

A cross-sectional descriptive study was carried out in Tenerife. A convenience sample was recruited. Health professionals (HPs) included doctors, nurses, psychologists, nursing assistants, and health science teachers from two large university hospitals, as well as urban primary care centers in the two main regions of the island. Health science students (HS) included those in nursing, medicine, and other health care training programs. Participants with other occupations (OO) were administrative, cleaning, and maintenance staff; people recruited in the waiting rooms of hospitals, outpatient clinics, primary care health centers, and teaching and administrative centers; and university students from non-health sciences and students from vocational training centers for young people and adults. Participants were informed about the study, informing the researcher if they wanted to participate or not. Data were collected through an anonymous, self-administered, and paper-based questionnaire that lasted an average of 30 min. The study was reviewed and evaluated by the Ethics Committee of the Canary Islands Public Health Service (CHUNSC_2019_12).

### 2.1. Measures

Sociodemographic variables included age, education (university, secondary or primary studies), occupation (health professionals (HP)s, health students (HSs), other occupations (OO)), biological sex (woman/man), comfort with assigned gender (yes/no), sexual orientation (heterosexual, gay, lesbian, bisexual, pansexual), and knowing someone (friend or acquaintance) transgender (yes/no).

A short version of the Genderism and Transphobia Scale (GTS; 12 items) was used that had been developed in Spain by Carrera-Fernández et al. [20], derived from the original English 32-item scale [4]. Items are scored on a scale of 1 to 7, from “completely disagree” to “completely agree”. It has 2 subscales with 6 items each (scores range between 6 and 42): transphobia/genderism (TG) assesses negative emotional reactions and attitudes toward trans* people, whereas gender bashing (GB) represents the behavioral component of transphobia, including a range of behaviors from taunting or verbal abuse to physical assault, which is a criminal offence. In the validation study with Spanish adolescents, Cronbach’s α was 0.83 for TG and 0.80 for GB and they showed good factorial and convergent validity [20].

These two subscales were empirically derived in the original validation studies by means of factor analysis and they do not differentiate between transphobia towards transgender men or women. Therefore, we constructed two theoretical scales to assess specific attitudes toward men (GTS_FtM_) and women (GTS_MtF_), separately. These two scales also have 6 items each: 5 from the short-form GTS used in this study and 1 from the original 32-item scale (2 items from the short-form GTS are not gender-specific).

The Negative Attitude towards Trans* people Scale (known in Spanish as EANT) is a 9-item scale developed in Argentina [21] with students and the general population. Items are scored on a scale of 1 to 5, from “completely disagree” to “completely agree” (scores range between 9 and 45). It is a monofactorial scale with good psychometric properties.

### 2.2. Statistical Analysis

Participants with more than four and two missed items on the GTS and EANT, respectively, were excluded from the analyses. For the remaining participants, missed values for each subject were substituted by the mean of the remaining items. 

Univariate associations of independent variables with the genderism/transphobia scales were assessed by means of linear regression for binary variables and analysis of variance (ANOVA) for non-binary variables. Multivariate linear regression models were used to assess the independent effect of correlates on the genderism/transphobia scales.

Finally, to assess the effect of the target (transgender women or men) and its interaction with participants’ biological sex, three ANCOVAs were made, one for each occupation group, using the GTS_FtM_ and GTS_MtF_ subscales as the intra-subject factor. 

## 3. Results

Seven hundred people were invited to participate, 632 agreed, and 624 completed the surveys. Twenty-two participants were excluded because they showed more than four or two missed items in the GTS and EANT, respectively, leaving a final analyzed sample of 602 participants (Table 1). 

The mean participant age was 34.60 years (SD = 12.90), 59% had completed some university education, 79% were women (this is representative of health students and health professionals in the Canary Islands where the majority are women), 10.5% were non-heterosexual and almost all participants (98.8%) were comfortable with their assigned sex. Approximately half of the sample (52.2%) stated that they knew a transgender person (Table 1).

Significant differences appeared when comparing the different groups: health students were the youngest, health professionals included more women and heterosexual participants, and people with other occupations were less educated and included more participants who knew a transgender person.

Cronbach’s α was 0.78 for TG and 0.70 for both GB and EANT. The GTS_FtM_ and GTS_MtF_ scales, though not empirically derived, showed acceptable α values (0.76 and 0.74, respectively). Mean scores were 10.0 (SD = 5.73) for TG, 8.02 (SD = 3.51) for GB, and 15.80 (SD = 4.58) for EANT.

### Correlates of Genderism/Transphobia

Univariate and multivariate correlates for the three scales were analyzed. At the univariate level, TG was significantly associated with all the independent variables. Health students scored significantly lower than the OO group (*p* = 0.006), with HH in the middle and not significantly different from the other two groups. In the multivariate model, this association remained significant (β = −0.10; *p* = 0.045). Being a man (β = −0.14; *p* = 0.001), heterosexual (β = −0.12; *p* = 0.003), and not knowing someone transgender (β = −0.12; *p* = 0.003) were significantly associated with higher scores. For BG, the only significant multivariate predictor was being a man (β = −0.14; *p* = 0.001). EANT was significantly predicted by older age (β = 0.22; *p* < 0.001), being a man (β = −0.09; *p* = 0.036), being heterosexual (β = −0.11; *p* = 0.007), and not knowing someone transgender (β = −0.11; *p* = 0.005). Education was significant at the univariate level for the three variables but not in the multivariate models.

How men and women value trans* people depending on whether these were transgender men (GTS_FtM_) or women (GTS_MtF_) was analyzed separately for each occupation. Repeated measures ANCOVA (adjusting for age) yielded no significant results for the effect of target in any subsample; that is, transgender men and women were not valued differently. However, the interaction between target and biological sex of the participants was significant for the HS and OO groups (in HP, the *p*-value was 0.088). Male participants were more transphobic toward transgender women, whereas the opposite occurred with female participants. However, post hoc comparisons (*t*-test for repeated measures) were significant only for female HS.

## 4. Discussion

This study aimed to assess and compare the attitudes toward trans* people among nurses and other health professionals, nursing and other health students, and a population not related to health service provision. We used two different scales, one originally developed in English (GTS) and the other in Spanish (EANT), which showed acceptable internal consistency. The EANT, recently developed in Argentina, has been less frequently used.

Our results showed low mean values of explicit prejudice/transphobia, especially in the GB behavioral dimension. Although a direct comparison with other studies is not straightforward due to the use of different scales or versions of the same scale, the observed level of transphobia is similar or even lower than that obtained in other countries [22,23,24]. As an average result, it is not surprising to find tolerant attitudes toward trans* people in liberal democracies, since, in the last few decades, social and political movements in these countries have denounced discrimination against minority groups. However, this general assertion has to be nuanced for several reasons. First, we assessed explicit, conscious attitudes by means of self-report. The abovementioned current political and social culture that rejects discrimination might increase the risk of social desirability bias and many participants may not have shown their true attitudes. However, research has shown that implicit, unconscious attitudes may differ from explicit ones, showing a complex pattern of interactions with other persons’ characteristics [25]; nonetheless, the definition and mutual relationship of implicit and explicit attitudes, as well as their usefulness for predicting actual discriminatory behaviors, are still under discussion [26]. Furthermore, even if the actual average level of transphobia is low, a small percentage of health professionals with transphobic attitudes may considerably impact the perceptions and satisfaction of trans* people in the health system. Therefore, interventions to change transphobic attitudes are still necessary to completely normalize the health care provided to these citizens. Likewise, it is important to develop interventions aimed to improve resilience in trans* people and their/our allies.

We only found a significant difference among occupations, with health students showing a lower value of explicit transphobia than people in other occupations for the TG factor. This could suggest a better understanding and acceptance of trans* people in current health science academic formation but, given the small size of the difference and the fact that it was not observed in the EANT scale, this finding should be interpreted with caution. Very few previous studies have assessed explicit transphobia in health professionals and most of them have been performed with mental health care providers. Kanamori et al., in a sample of doctors, nurses, and other health professionals from the USA, did not find significant differences compared with a normative group in the three subscales of the scale used [27]. Fisher et al. found that among heterosexuals, female health professionals were less transphobic than their male counterparts and male or female controls [5]. In the field of mental health, a recent systematic review by Brown et al. included 13 studies that showed, in general, an overall positive attitude toward trans* people [28]. Mental health providers may have better formation and more experience working with trans* persons and may therefore show less transphobia than other health professionals but there are no studies directly comparing these populations. 

The significant correlates of transphobia obtained in this study mostly confirm previous findings [5,10], showing an association with a normative sociodemographic profile: being older (only for the EANT scale), male, heterosexual, and not knowing a transgender person. Interestingly, education was not significantly related to transphobia in the multivariate models, indicating that a high education level is not a protective factor against prejudice. As mentioned above, interventions to change attitudes are still needed. The result that not knowing transgender people significantly relates to transphobia supports the hypothesis that contact with trans* people could be a successful way to mitigate these attitudes [29,30].

Several studies indicate that nearly 50% of surveyed health science professionals report a lack of knowledge about the needs of LGBTI people [31,32]. Therefore, although most professionals do not share negative attitudes, the need for accurate training is widespread because the quality of health care is not only indicated by the absence of prejudice. More knowledge about trans-specific health care is needed in both specialized care centers and general health care. In addition, the perception of transphobia, fragmentation of services (i.e., the person is not treated holistically, but is treated as divided into parts or pathologies such as respiratory and endocrinology; in the case of trans* people, this finding is worse), administrative issues, and the lack of cultural sensitivity of professionals are considered barriers to care [3]. Education of future health professionals is essential to improve health care provision and outcomes for trans* people. A recent systematic review showed that although the incorporation of transgender health in medical curricula has increased internationally in recent years, there is still no consensus about the most appropriate and effective educational training [33].

This study has several limitations. We have already mentioned the self-reported nature of the assessment, with the subsequent risk of desirability bias; therefore, the results must be interpreted in the context of explicit attitudes. Regarding generalizability, the use of a non-randomly selected sample could introduce selection bias; there were significant differences among the three samples in several independent variables.

## 5. Conclusions

We found low mean values of explicit transphobia and negative attitudes, with little or no difference between health care workers or students and those in other occupations. The correlates of transphobia confirm previous results, except that both men and women are less transphobic toward trans* persons whose identities coincide with their own. Although most professionals do not explicitly show transphobic attitudes, they could maintain unconscious prejudice, which may translate to discriminatory behaviors when they interact with trans* people. In professionals who do not share transphobic attitudes, the barriers identified by trans* people might be a problem due to the lack of specific training.

## Figures and Tables

**Table 1:** Characteristics of the sample.

Variables	Health Professionals (HP) (*n* = 159)	Health Students (HS) (*n* = 212)	Other Occupations (OO) (*n* = 231)	^a^ *p*
Age (mean, SD)	40.80 (9.48)	25.90 (9.27)	38.40 (13.8)	<0.001
Education				<0.001
University	116 (73.0%)	149 (70.3%)	90 (39.0%)	
Secondary	40 (25.2%)	47 (22.2%)	113 (48.9%)	
Primary	3 (1.9%)	16 (7.5%)	28 (12.1%)	
Biological sex (% of women)	129 (81.6%)	148 (70.5%)	135 (58.4%)	<0.001
Gay, lesbian, bisexual, or pansexual	7 (4.5%)	26 (12.4%)	29 (12.6%)	0.018
Comfort with assigned gender	159 (100%)	211 (99.5%)	225 (97.4%)	0.372
Know a transgender person	76 (47.8%)	102 (48.1%)	136 (58.9%)	0.034

^a^*p*-value from ANOVA for age; χ^2^ test for the remaining variables.
